# Anodal transcranial direct current stimulation of the right anterior temporal lobe did not significantly affect verbal insight

**DOI:** 10.1371/journal.pone.0184749

**Published:** 2017-09-13

**Authors:** Takatsugu Aihara, Takeshi Ogawa, Takeaki Shimokawa, Okito Yamashita

**Affiliations:** 1 Neural Information Analysis Laboratories, Advanced Telecommunications Research Institute International (ATR), Kyoto, Japan; 2 Cognitive Mechanisms Laboratories, Advanced Telecommunications Research Institute International (ATR), Kyoto, Japan; University Medical Center Goettingen, GERMANY

## Abstract

Humans often utilize past experience to solve difficult problems. However, if past experience is insufficient to solve a problem, solvers may reach an impasse. Insight can be valuable for breaking an impasse, enabling the reinterpretation or re-representation of a problem. Previous studies using between-subjects designs have revealed a causal relationship between the anterior temporal lobes (ATLs) and non-verbal insight, by enhancing the right ATL while inhibiting the left ATL using transcranial direct current stimulation (tDCS). In addition, neuroimaging studies have reported a correlation between right ATL activity and verbal insight. Based on these findings, we hypothesized that the right ATL is causally related to both non-verbal and verbal insight. To test this hypothesis, we conducted an experiment with 66 subjects using a within-subjects design, which typically has greater statistical power than a between-subjects design. Subjects participated in tDCS experiments across 2 days, in which they solved both non-verbal and verbal insight problems under active or sham stimulation conditions. To dissociate the effects of right ATL stimulation from those of left ATL stimulation, we used two montage types; anodal tDCS of the right ATL together with cathodal tDCS of the left ATL (stimulating both ATLs) and anodal tDCS of the right ATL with cathodal tDCS of the left cheek (stimulating only the right ATL). The montage used was counterbalanced across subjects. Statistical analyses revealed that, regardless of the montage type, there were no significant differences between the active and sham conditions for either verbal or non-verbal insight, although the finding for non-verbal insight was inconclusive because of a lack of statistical power. These results failed to support previous findings suggesting that the right ATL is the central locus of insight.

## Introduction

During problem solving, we typically attempt to find solutions based on prior experience of solving similar problems. In such cases, solutions are often reached through step-by-step, analytical processes that do not involve insight. Analytical solutions are a product of conscious processing, and problem solvers can explain how they reached a solution. In some situations, however, a problem cannot be solved using conventional stepwise methods alone, and require the use of insight. When faced with particularly difficult problems, it is common to attempt all known strategies before reaching an impasse, then to stop attempting to find a solution because all strategies have been exhausted [1]. After a period of incubation, insight may suddenly occur, sometimes accompanied by an “Aha!” experience. At this time, the problem solver may reinterpret or re-represent the problem by relaxing self-imposed constraints and/or decomposing chunked items in the problem [2, 3]. A solution may then be reached by re-applying conventional stepwise methods. Insights are largely a product of unconscious processing [4]; problem solvers typically cannot report the processing that enabled them to suddenly overcome an impasse and reach a solution [5].

Research on insight has a long history. However, the neural mechanisms underlying the phenomenon remain poorly understood. Bowden and Jung-Beeman conducted behavioral experiments to examine hemispheric differences in the information processing involved in solving insight problems, using the compound remote-associate (CRA) test as a verbal insight problem [5]. In the CRA test, subjects are asked to generate a fourth word that would form a compound word with each of three problem words (e.g., *high/district/house—school*). The results revealed that subjects showed greater priming (i.e., response latencies showed greater decreases) for solution words presented to the left visual field (i.e., the right hemisphere; RH) compared with those presented to the right visual field (i.e., the left hemisphere; LH). These results suggest that, in a problem-solving context, there was greater activation of solution-relevant information in the RH than in the LH. These RH advantages occurred only when problem solvers experienced insight [6]. To provide direct physiological evidence for the neural mechanisms underlying insight, Jung-Beeman and colleagues then conducted functional magnetic resonance imaging (fMRI) and electroencephalography (EEG) experiments while subjects performed the CRA test [7, 8]. Jung-Beeman et al. [7] reported that fMRI data (collected with a 1.5 Tesla scanner) revealed increased activity in the RH anterior superior temporal gyrus for insight-related relative to non-insight-related solutions, and that EEG data revealed a sudden burst of high-frequency (gamma-band) neural activity in the same area, beginning 0.3 s prior to insight solutions. In addition, fMRI data revealed that weak (i.e., subthreshold) activity was observed in other areas, including bilateral hippocampus and parahippocampal gyri, as well as anterior and posterior cingulate cortex (ACC and PCC). This result was replicated by Subramaniam et al. [8] with more subjects and better imaging methods, using a 3 Tesla MRI scanner. Activation in all of these areas reached statistical significance, with the RH anterior temporal region again displaying the strongest activation. Furthermore, Kounios et al. [9] recorded high-density EEG while subjects solved a series of anagrams (also a verbal insight problem) to compare resting-state or preparatory brain activity between insight and analytical solutions. The results revealed that insightful individuals showed greater RH activity at rest than analytic individuals. A range of studies by Jung-Beeman and colleagues suggest RH dominance for insight, at least in the verbal domain [5,6,7,8,9].

The neuroimaging findings discussed above demonstrate only a correlational relationship between RH (particularly the right anterior temporal region) activity and insight-related solutions. Proving a causal relationship requires evidence that selective manipulation of RH activity alters performance on insight problems. Transcranial direct current stimulation (tDCS) is a non-invasive brain stimulation technique that can be used to manipulate brain activity. To our knowledge, only two previous tDCS studies have examined the causal relationship between RH anterior temporal region activity and insight solutions [10, 11]. Both studies proposed that the LH is involved in the maintenance of existing hypotheses and representations, while the RH is associated with novelty, as well as updating hypotheses and representations. Moreover, it was hypothesized that cathodal stimulation (decreasing excitability) of the left anterior temporal lobe (ATL) together with anodal stimulation (increasing excitability) of the right ATL would facilitate performance on insight problems. The results of both studies supported the hypothesis, using between-subjects designs involving non-verbal insight problems, including the matchstick arithmetic task [10] (see below for a description) and the nine-dot problem [11]. On the basis of these findings, together with the findings of the neuroimaging studies using verbal insight problems (i.e., the CRA and anagram) mentioned above, we hypothesized that the right ATL is causally related to insight in general (i.e., both verbal and non-verbal insight). To our knowledge, at least four previous tDCS studies have examined verbal insight. Three studies [12, 13, 14] targeted the left dorsolateral prefrontal cortex (DLPFC), whereas one study by Goel et al. [15] targeted the right temporo-parietal junction (TPJ). Although each of these studies demonstrated that anodal tDCS to each target resulted in increased performance, no study has demonstrated that anodal tDCS to the right ATL facilitates verbal insight. In the present study, we examined whether anodal tDCS to the right ATL facilitates verbal as well as non-verbal insight, using an experimental paradigm similar to that used by a previous study by Chi and Snyder [10]. In addition, we extended this previous study [10] to examine two additional issues. First, we examined whether anodal tDCS of the right ATL facilitates insight within subjects as well as between subjects. Importantly, because between-subjects variability is typically greater than within-subjects variability, within-subjects designs often have greater statistical power than between-subjects designs, making them more likely to correctly reject a null hypothesis. Second, we examined specific effects of right ATL stimulation using an additional tDCS electrode montage (the “extra-cephalic” reference montage) in which a cathode was placed on the left cheek (having no effect on the LH brain) while the anode was placed over the right ATL; note that the “bi-cephalic” electrode montage (anode over the right ATL together with cathode over the left ATL) used in the previous study [10] stimulated both ATLs. In addition, it is possible to infer the effects of left ATL stimulation on insight by comparing the combined effects of right and left ATL stimulation with a “bi-cephalic” montage and the specific effects of right ATL stimulation with an “extra-cephalic” montage, if the effects of anodal and cathodal tDCS are assumed to be additive. It should be noted that, throughout this study, we have interpreted the effects of left ATL stimulation based on this assumption.

Most insight research in the last several decades has used “classic” insight problems involving a small collection of verbal riddles and spatial puzzles, such as the 9-dot problem [1]. Because these classic insight problems are limited in number and vary widely in mode of presentation, category (verbal, mathematical and spatial) of insight needed, and level of difficulty, the research field of insight has lacked large sets of homogenous stimuli. This lack of homogenous stimuli sets makes it difficult to compare insight problem performance across experiments (e.g., active tDCS condition vs. sham condition) using classic problems.

To overcome the limitations of classic problems, researchers have developed new insight problems with two particularly useful properties: first, the experimenter can develop a large number of relatively homogenous problems; second, the experimenter can manipulate the level of difficulty of the problems [1]. Among the “new” insight problems is the remote associate test (RAT), in which participants are asked to generate a solution word associated with each of the three problem words (e.g., *same/tennis/head—match*) [16]. The other prominent “new” insight problem is the matchstick arithmetic task in which participants are asked to solve the equation by moving only one matchstick (e.g., IV = III + III requires the “I” before the “V” to be placed after the “V”, creating VI = III + III) [17]. Although these new insight problems may not be as complex as classic insight problems, they exhibit three properties of insight problems that distinguish insight from non-insight solutions [3, 16]. First, solvers often take the incorrect course and reach an impasse. Second, solvers typically cannot report how they overcame the impasse. Third, solvers often have an Aha! experience when they achieve correct solutions. Moreover, one study reported that performance on the RAT reliably correlated with success on classic insight problems [3]. Thus, solving these new insight problems appears to involve the same component processes critical for “classic” insight problems [16]. In the present study, we used a variant of the RAT as a verbal insight problem and a variant of the matchstick arithmetic task as a non-verbal insight problem (see Materials and methods for detail).

## Materials and methods

**Fig 1:**
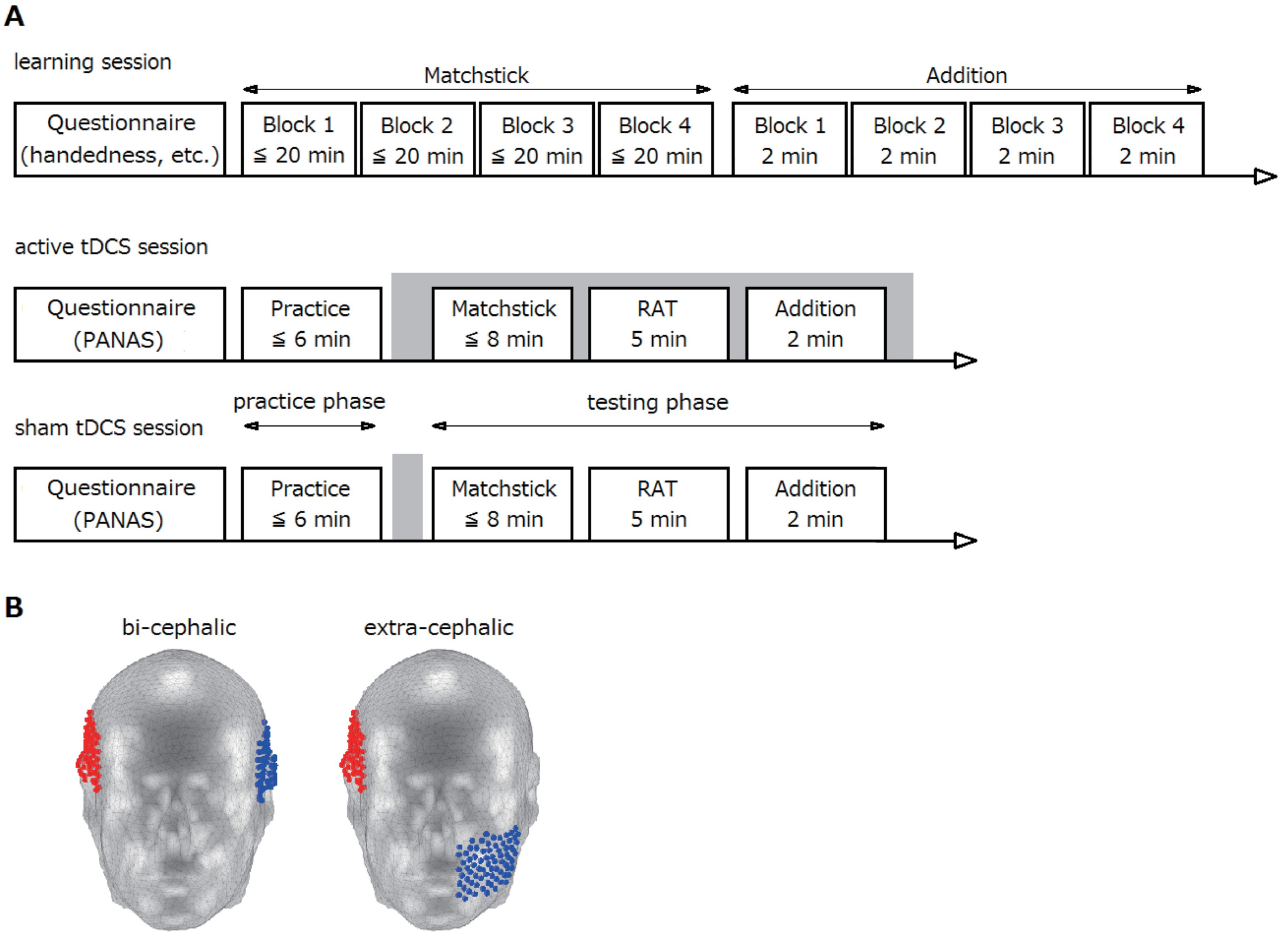
Experimental procedure and tDCS electrode montages. (A) A schematic representation of the experimental procedure. The shaded area represents the time of tDCS application. Each subject completed all three sessions on separate days; the first tDCS session was conducted 1–4 days after the learning session, and the two tDCS sessions were separated by an interval of 1 week (more than 1 week for a few subjects). The order of active and sham tDCS sessions was counterbalanced across subjects to reduce the possibility of any potential learning effects. In tDCS sessions, the testing phase started 5 min after tDCS onset to ensure that there was sufficient change in cortical excitability [10]. (B) tDCS electrode montages. Left, the bi-cephalic electrode montage. Right, the extra-cephalic reference electrode montage. The anodal and cathodal electrodes are shown in red and blue, respectively. These figures were drawn using the COMETS toolbox.

Each subject completed one learning session and two tDCS sessions, each of which is illustrated in Fig 1A. Details of the experiment are described in each subsection below.

### Subjects

Sixty-six subjects (53 males) aged between 18 and 44 participated, in return for money. All were native Japanese speakers and reported no history of neurological or psychiatric disorders. All subjects had normal or corrected to normal vision. All subjects gave written informed consent to participate in the experimental procedures, which were approved by the ATR Review Board Ethics Committee. Of the 66 subjects, one subject who participated in only the learning session and two subjects who were left-handed according to the Edinburgh Handedness Inventory [18] were excluded from the analysis. The remaining 63 subjects were divided into two groups (see Table 1 for demographic characteristics) according to the electrode montage condition (see the Transcranial direct current stimulation (tDCS) subsection and Fig 1B for the electrode montage).

**Table 1:** Demographic characteristics across the two montage groups.

	“Bi-cephalic”	“Extra-cephalic”
**Age**	29.3 ± 9.6	25.5 ± 7.5
**N (male / female)**	32 (24 / 8)	31 (26 / 5)
**Handedness score**	92.8 ± 17.0	94.9 ± 9.0

Values in the first (age) and third (handedness score) rows are presented as mean ± standard deviation (SD). Values in the second row show the number of subjects (males/females). Between the two montage groups, subjects did not differ in terms of age (p = 0.08, two-sample t-test), gender (p = 0.29, Fisher’s exact test) or handedness score (p = 0.55, two-sample t-test). “Bi-cephalic” = Bi-cephalic electrode montage group; “Extra-cephalic” = Extra-cephalic reference montage group.

### Transcranial direct current stimulation (tDCS)

tDCS is a non-invasive brain stimulation technique in which weak, constant electric currents are applied to the scalp to alter cortical excitability by shifting the resting membrane potential. Depending on current flow direction relative to neuronal orientation, tDCS depolarizes (anodal stimulation) or hyperpolarizes (cathodal stimulation) cortical neurons at a subthreshold level, thereby modulating spontaneous firing frequency [19].

In the current study, stimulation was delivered by a battery-driven electrical stimulator (DC-Stimulator Plus II; neuroConn GmbH, Ilmenau, Germany) through conductive-rubber electrodes (7 × 5 cm). Both the anode and cathode were covered with saline-soaked sponges for the bi-cephalic electrode montage; the anode was covered with saline-soaked sponge and the cathode was covered with Ten20 conductive paste (Weaver and company, Aurora USA) for the extra-cephalic reference montage. The anode was placed above the right ATL with the center of the electrode positioned at 1.5 cm anterior to T4 (according to the international 10–20 system). The cathode was placed over the left ATL with the center of the electrode positioned 1.5 cm anterior to T3 for the bi-cephalic electrode montage. For the extra-cephalic reference montage, the cathode was placed on the left cheek (Fig 1B). Before adopting these electrode montages, we conducted preliminary computer simulations (see Supporting Information for details) using the COMETS [20], a MATLAB toolbox for simulating local electric fields generated by tDCS based on the electrostatic finite element method (FEM). In simulations with a standard human head model provided in the toolbox, we confirmed that tDCS with the bi-cephalic electrode montage (anode/cathode over the right/left ATLs, respectively) generates electrical fields in widespread temporal areas including bilateral ATLs. In contrast, tDCS with the extra-cephalic reference montage (the anode over the right ATL and the cathode on the left cheek) generates similar electric fields in the RH, whereas almost no electric fields can be observed in the LH.

For the active stimulation session, a constant current was applied for 25 min (with 15-sec ramp up/down) at 1.6 mA intensity (current density = 0.0457 mA/cm^2^). For the sham stimulation session, the electrodes were placed in the same positions as in the active stimulation session, but stimulation lasted only 2 min, so that subjects did not receive further stimulation for the rest of the experiment. Previous studies have established that short stimulation lasting less than 2 min generates cortical excitability alterations only during stimulation, while longer-lasting tDCS (longer than 10 min) induces sustained (1 h or longer) excitability alterations [19, 21, 22]. Because the first task (the matchstick arithmetic task; see below) started approximately 3 min after the end of stimulation (see the Procedure subsection and Fig 1A for the experimental schedule), tDCS would not be expected to affect task performance in the sham stimulation session.

After the second tDCS session, 24 subjects were asked to judge which tDCS session yielded a stronger sensation of stimulation.

### Non-verbal insight problems—Matchstick arithmetic task

**Fig 2:**
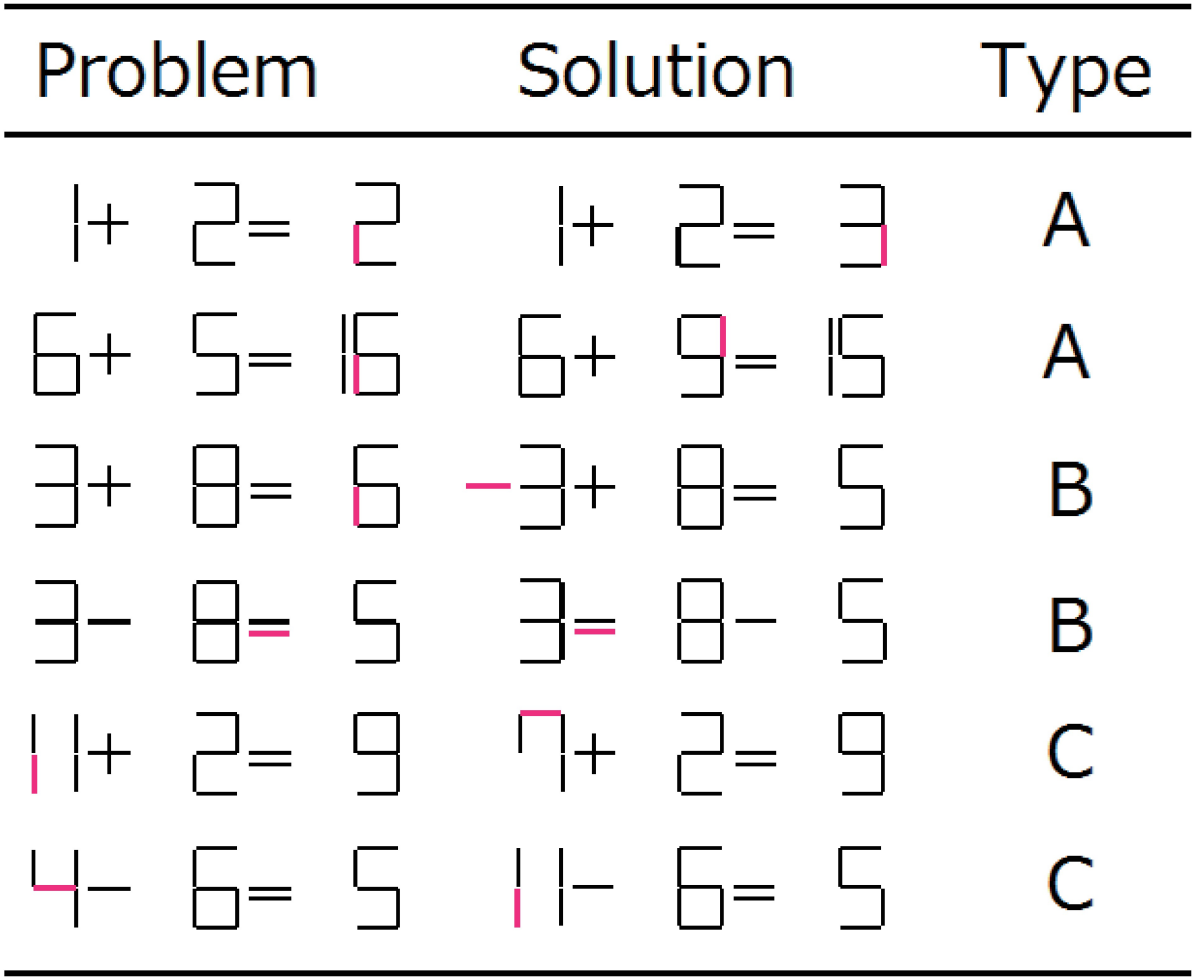
An example of the non-verbal insight problems used in the matchstick arithmetic task. Column one displays the problem, and column two gives the corresponding solution. The last column indicates the problem type. The magenta lines in columns one and two indicate the sites where manipulation occurred. Type-A insight problems were used in the learning session and practice phase of the tDCS session. Type-B and Type-C problems were used in the testing phase of the tDCS sessions.

To assess whether tDCS facilitates non-verbal insight, we used the matchstick arithmetic task consisting of Arabic numerals (see Fig 2 for examples) because Roman numerals, which were used in previous studies [2, 10], are rare in Japan. In this task, subjects were instructed to identify the correct arithmetic statement (the solution) by moving a stick from one position to another position without adding or discarding sticks. Subjects were instructed not to use the following three signs; ‘≠’, ‘<‘, and ‘>‘. The matchstick problem can be divided into the three types: Type-A, Type-B and Type-C problems.

In the matchstick arithmetic task, a standard Type-A problem requires changes to only numerical characters (values). For example, 1 + 2 = 2 requires the subject to change 2 on the right-hand side to 3, producing the solution 1 + 2 = 3 (see the first row of Fig 2). Note that this transformation requires the subject to move a stick within a numerical character. As another example, 6 + 5 = 16 requires moving a stick from one numeral (in this case, 6 on the right-hand side) to another (in this case, 5), producing the solution 6 + 9 = 15 (see the second row of Fig 2).

A Type-B problem requires a change to a sign or operator. For example, 3 + 8 = 6 requires the subject to move a stick from 6 in the right-hand side to the leftmost position to yield the minus sign (‘−’), leading to the solution −3 + 8 = 5 (see the third row of Fig 2). Another example, 3 − 8 = 5 requires interchanging one horizontal stick between the equal sign (‘ = ‘) and minus sign (‘−’) to obtain 3 = 8 − 5 (see the fourth row of Fig 2).

A Type-C problem requires changing a two-digit number to a one-digit number, and vice versa. For example, 11 + 2 = 9 requires changing 11 (two-digit number) to 7 (one-digit number), producing the solution 7 + 2 = 9 (see the fifth row of Fig 2). As another example, 4–6 = 5 requires changing 4 (one-digit number) to 11 (two-digit number), producing the solution 11–6 = 5 (see the sixth row of Fig 2). Type-C problems are a particular type of Type-A problem, because they also require changes to only numerical characters.

According to Ollinger et al. [17], if a subject is repeatedly presented with problems that are all solved by the same solution strategy (e.g., Type-A problems), they tend to reach an impasse on a subsequent problem requiring a different solution strategy (e.g., a Type-B problem). In such a case, the problem solver will be able to reach a solution only if they can break an impasse using insight [4]. The present study was designed to enable subjects to solve problems using insight in the testing phase (see the Procedure subsection and Fig 1A for the testing phase) of the tDCS session. As such, only Type-A problems were used in the learning session and the practice phase (see the Procedure subsection and Fig 1A for the practice phase) of the tDCS sessions, and Type-B and C problems were used in the testing phase of the tDCS sessions. There are 24 possible changes from a one-digit number to another one-digit number with the constraint that solvers can move only one stick. We created 19 Type-A problems, covering all 24 possible changes. In the learning session, subjects were given all 19 Type-A problems in each block; the order of the problems was randomized across blocks and subjects. Because each subject underwent four blocks, they solved the same problem four times. Thus, we expected that subjects would learn how to solve Type-A problems.

### Verbal insight problems—RAT task

**Fig 3:**
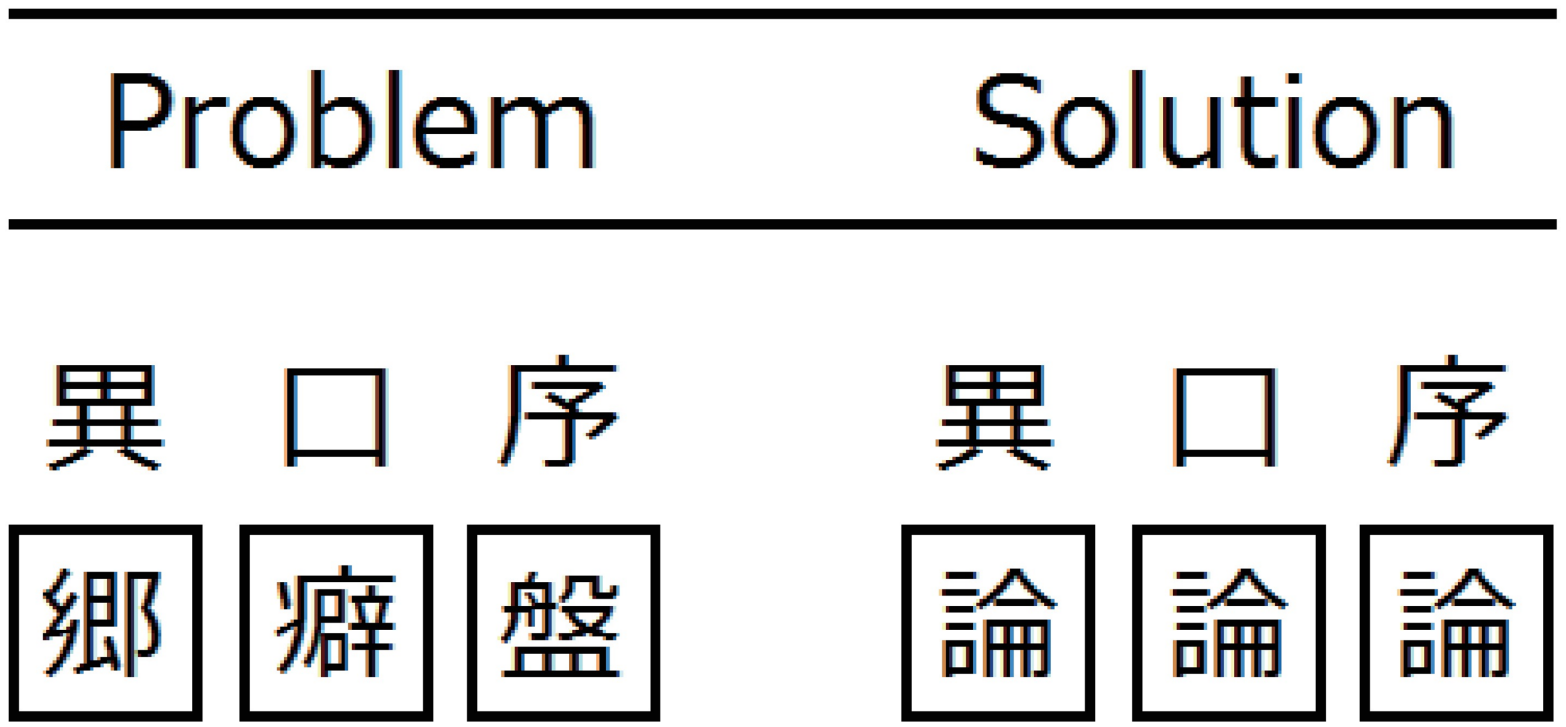
An example of the verbal insight problem (the RAT task). Column one displays the problem. Column two gives the corresponding solution. English translations of each two-word phrase are as follows: 異郷 (strange land), 口癖 (habitual saying), 序盤 (initial phase), 異論 (different opinion), 口論 (dispute), 序論 (introduction).

To assess whether tDCS can facilitate verbal insight, we used the Japanese version of the RAT task (Fig 3) developed and validated by Terai and colleagues [23]. Participants were asked to find the correct kanji to form a compound word or two-word phrase with each of the three upper kanjis. Each kanji in the square is called a “filler”, which forms a two-word phase with each of the upper kanjis and serves as a distracter. Such fillers are designed to hinder subjects from finding correct answers and to cause an impasse, prompting them to solve the problem with insight. It should be noted that the standard RAT task without distracters is not always solved with insight; for example, a previous study reported that approximately 60% of problems were solved with insight, while approximately 40% were solved without insight [7].

Of 79 Japanese RAT problems, five were chosen for practice phase, and two different sets of 20 problems were chosen for testing phase (see the Procedure subsection for practice and testing phases), counterbalanced across tDCS conditions (sham vs. active). The difficulty of problem sets was based on data provided by Terai et al. [23].

### Non-insight problems—Two-digit addition arithmetic task

Participants were also given a simple arithmetic task in which they answered as many two-digit addition arithmetic problems (e.g., 49 + 17 = ?) as possible in 2 min. This task did not require any insight and served as a control task.

Lesion studies revealed that disturbances in arithmetical calculation are often observed after damage to the left inferior parietal lobe [24, 25]. Repetitive transcranial magnetic stimulation (rTMS) over left inferior parietal areas, but not over the right side homologue, disrupted performance of double-digit addition tasks [26]. In addition, bilateral tDCS with the anode over the left parietal cortex and the cathode over the right parietal cortex was found to shorten response times of the mental calculation tasks (a multiplication problem) in some subjects [27]. These studies strongly suggest that the (inferior) parietal cortex is important for calculation. In addition, a quantitative meta-analysis of fMRI studies revealed that multiple areas such as the precuneus, cerebellum, frontal and prefrontal areas and parietal areas were activated during calculation tasks (addition, multiplication, subtraction), but these regions did not include the ATL or surrounding areas [28]. Thus, we assumed that the ATL is not associated with calculation, and, therefore, that the two-digit addition arithmetic task is a suitable control task.

### Procedure

We used a within-subjects design in which each subject completed one learning session and two tDCS sessions on three different days (Fig 1A). The two tDCS sessions were for active and sham tDCS conditions, the order of which was counterbalanced across subjects. The first tDCS session was conducted 1–4 days after the learning session. The two tDCS sessions were spaced at least 1 week apart and were conducted at the same time of day to avoid the possible effects of diurnally structured variation. Subjects were blind to the stimulation condition, and were informed only that there were two tDCS sessions separated by 1 week.

We conducted the learning session for two reasons. First, the present study used a within-subjects design in which each subject participated in active and sham tDCS sessions on two different days. Thus, it was important to reduce the possibility that learning effects would distort the results. In preliminary experiments consisting of active and sham tDCS sessions (but not a learning session), we found that performance on the matchstick and two-digit addition arithmetic tasks (but not the RAT task) was higher on day 2 than day 1, suggesting that learning effects occurred in the two tasks. For this reason, we conducted learning sessions for both the matchstick and two-digit addition arithmetic tasks, and removed subjects whose performance did not saturate during the learning session from the analysis. Second, as described above (see the Non-verbal insight problems subsection), in the matchstick arithmetic task, learning one solution strategy is expected to enhance the probability of solving problems with insight if other solution strategies are required.

A maximum of four subjects participated in the learning session at the same time. They sat on comfortable chairs in a quiet room, facing their computer monitors. There were partitions so that subjects could not see the monitors used by the other subjects.

The learning session was conducted in the following way (Fig 1A). First, subjects were asked to complete questionnaires on their handedness (Edinburgh handedness inventory [18]) and medical history of neurological or psychiatric disorders. Next, they received instructions about the matchstick arithmetic task and performed four blocks of the task. They were then given instructions about the two-digit addition arithmetic task and performed four blocks of the task. Finally, they completed a questionnaire regarding sleep (duration and quality), intake of alcohol/caffeine/food/drugs, and prior experience of the matchstick arithmetic task (We did not use these data in the present study).

Subjects took part in one tDCS session at a time. They sat on a comfortable chair in a quiet room, facing a computer monitor.

The tDCS sessions were performed in the following way (Fig 1A). First, subjects were asked to complete the Japanese version of the positive and negative affect scales (PANAS [29, 30]). The electrodes were then attached to their scalp by the experimenters and held in place by rubber bands. After the tDCS-setup was completed, subjects received instructions for the matchstick arithmetic, RAT, and two-digit addition arithmetic tasks and practiced each task briefly (within a total period of 6 min; practice phase). After the practice phase, brief instructions were given about the testing phase, and electrical stimulation began. Approximately 5 minutes after tDCS started, subjects were asked to begin the testing phase, in which they completed the matchstick arithmetic, RAT, and two-digit addition arithmetic tasks, respectively. Finally, subjects completed a questionnaire consisting of questions regarding sleep (duration and quality), intake of alcohol/caffeine/food/drugs, and tDCS-induced sensations and phosphenes.

The problems used in the practice phase were different from those used in the learning session and testing phases of the tDCS sessions. It should be noted that, as mentioned above, the matchstick problems used in the practice phase consisted of only Type-A problems.

Regardless of task (matchstick/RAT/addition) and session (learning/tDCS), stimulus presentation and response collection were controlled using MATLAB 8.1 (MathWorks) with Psychtoolbox [31]. The refresh rate of the computer monitor was 60 Hz. Subjects were instructed to press the ENTER key as soon as they found the solution to each problem (the response time was defined as the time taken from the presentation of each problem to pressing the ENTER key; immediately after the key was pushed, the problem disappeared), then to type the answer accurately and press ENTER. When the time limit was exceeded (see below for the time limit of each task), if the problem had still not been answered, the problem disappeared. In the learning session and practice phase of the tDCS sessions, subjects were then given the correct solution and asked to press ENTER when they understood it. This step was skipped in the testing phase of the tDCS sessions. Subjects were then instructed to press ENTER to move to the next problem.

In the matchstick arithmetic task, subjects were asked to type the equation (e.g., 1 + 2 = 3 in the case of the uppermost problem of Fig 2) as the answer. In the RAT task, they were asked to type the Chinese-style reading of the solution kanji with alphabetical characters as the answer. In the two-digit addition arithmetic task, participants were asked to type the solution number (e.g., 66 in the case of 49 + 17 = ?) as the answer.

The time limit for each problem differed across tasks and sessions. In the matchstick arithmetic task, the time limits were 60 sec, 90 sec and 4 min for the learning session, practice phase and testing phase of the tDCS sessions, respectively. In the RAT task, the time limit was always 45 sec. In the two-digit addition arithmetic task, the time limit was always 5 sec.

For the matchstick arithmetic task, the number of problems in each block was fixed to 19 and 2 in the learning session and testing phase of the tDCS session, respectively, whereas it was not fixed in the practice phase of the tDCS session, in which subjects solved as many problems as possible within 2 min. For the RAT task, the number of problems in each block was not fixed in both the practice and testing phases of the tDCS sessions, in which subjects solved as many problems as possible within 1.5 min and 5 min, respectively. For the two-digit addition arithmetic task, the number of problems in each block was not fixed; subjects solved as many problems as possible within 2 min, 0.5 min and 2 min for each block of the learning session, practice and testing phases of the tDCS sessions, respectively. Thus, in the active tDCS session, it took less than 8 min to complete the matchstick task, 5 min to complete the RAT task and 2 min to complete the two-digit addition arithmetic task; consequently, it took less than 15 min (8 + 5 + 2 = 15) to complete all three tasks. Since subjects started the first task (i.e., matchstick) at least 5 min after tDCS started, they finished the last task (i.e., addition) approximately 20 min after tDCS started. Therefore, a 25 min duration of tDCS was sufficient for completing all three tasks (Fig 1A).

### Data analysis

For each task, we chose from the following three performance measures: the number of correct answers, proportion of correct answers, and response times for correct answers. The measures used in each task are described below.

In the learning session, all three measures were used for the two-digit addition arithmetic task, whereas only two measures, the proportion of correct answers and response time for correct answers, were used for the matchstick arithmetic task. The number of correct answers for the matchstick arithmetic task was excluded because it was positively correlated with the proportion of correct answers, since the number of matchstick problems in each block was fixed to 19.

In the testing phase of the tDCS session, two measures were used for the matchstick arithmetic task: the number of correct answers, and response time for correct answers. For both the RAT and two-digit addition tasks, the number and proportion of correct answers were used as performance measures. The proportion of correct answers was avoided for the matchstick arithmetic task because it was positively correlated with the number of correct answers, since the number of the matchstick problems was fixed to two. Response time for correct answers was avoided because the number of correct answers would partially reflect response speed.

For the data obtained in the learning session, one-way analysis of variance (ANOVA) and post-hoc Tukey’s HSD tests were used to investigate how the performance measures changed between blocks. For the data obtained in the tDCS session, paired sample t-tests were used to compare performance measures between active and sham tDCS sessions. Bias-corrected effect sizes (Hedges’ g) of active stimulation compared with sham stimulation and 95% confidence intervals (CIs) were then computed using the Effect Size Calculator (http://www.cem.org/effect-size-calculator). All statistical analyses except for the calculation of effect sizes were performed using the MATLAB Statistics toolbox.

## Results

### Learning session

#### Matchstick arithmetic task

**Fig 4:**
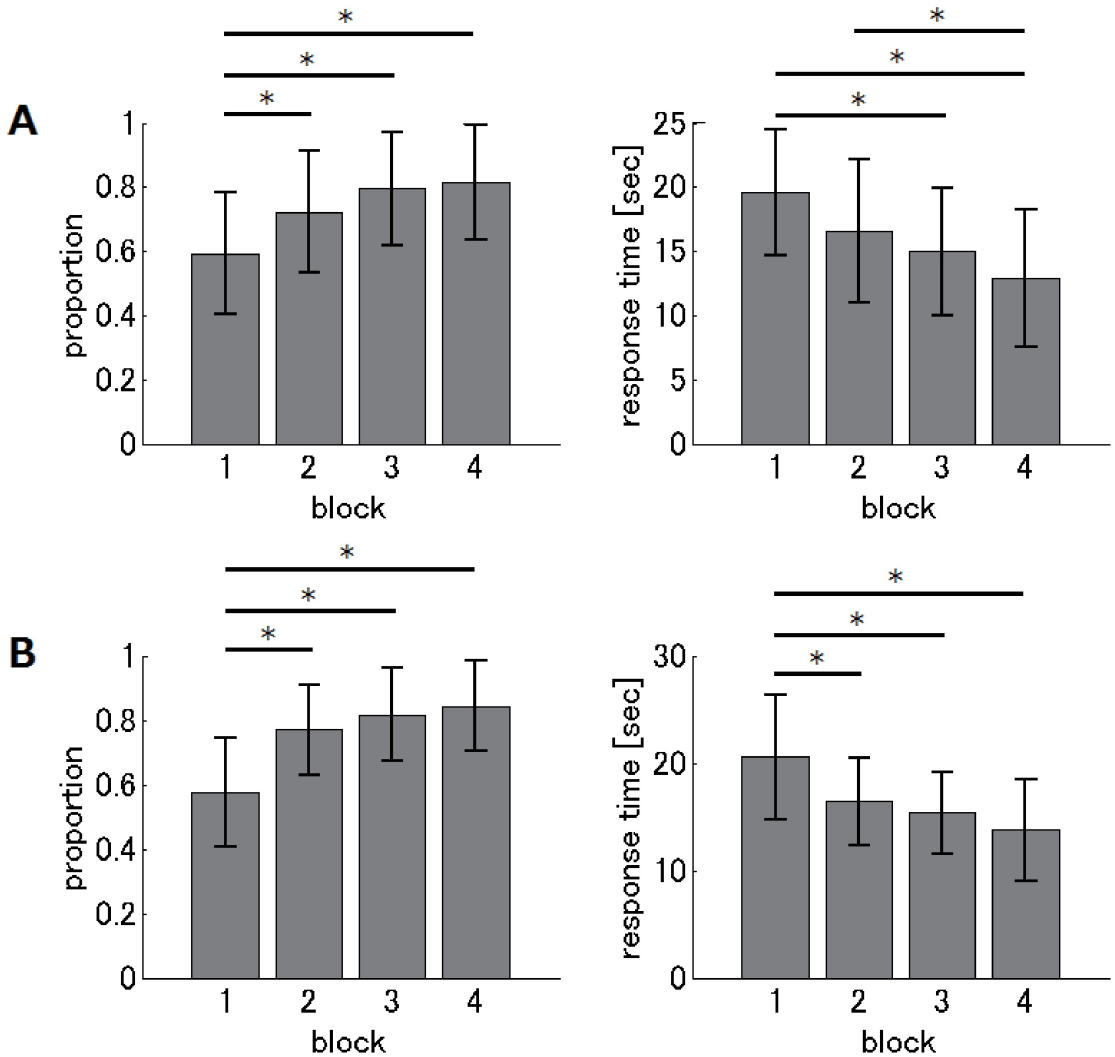
Results of the matchstick arithmetic task during the learning session. The left and right panels show the proportion of correct answers and response times for correct answers, respectively. Each gray column represents the mean (across subjects) value for each block. Error bars represent standard deviations. Asterisks indicate significant (p < 0.05) differences between blocks. (A) The “bi-cephalic” group. (B) The “extra-cephalic” group.

Fig 4 illustrates, for each montage group, how performance measures on the matchstick arithmetic task changed with the increased number of blocks in the learning session. We first describe the results of the bi-cephalic electrode montage group (“bi-cephalic” group; Fig 4A). One-way analysis of variance (ANOVA) revealed a significant difference for block in both performance measures (F_(3,124)_ = 9.66, p < 10^−5^ for the proportion of correct answers; F_(3,124)_ = 9.46, p < 10^−4^ for response time of correct answers). The post-hoc Tukey’s HSD test revealed that both performance measures in the first block were significantly different from those in the last two blocks (p < 0.05), and both measures were not significantly different between the last two blocks (p > 0.05), suggesting that performance substantially improved, then saturated. Three subjects did not reach 0.7 in the proportion of correct answers, indicating that they did not learn the task sufficiently, and were removed from subsequent analysis. Second, we describe the results of the extra-cephalic reference montage group (“extra-cephalic” group; Fig 4B). One-way ANOVA revealed significant differences between blocks in both performance measures (F_(3,120)_ = 20.4, p < 10^−10^ for proportion of correct answers; F_(3,120)_ = 12.02, p < 10^−6^ for response time of correct answers). The post-hoc Tukey’s HSD test showed that both measures in the first block differed from those in other blocks (p < 0.05), and both measures were not significantly different among the last three blocks (p > 0.05), suggesting that performance dramatically improved, then saturated. Three subjects did not reach 0.7 in the proportion of correct answers, indicating that they did not learn the task sufficiently. These subjects were removed from subsequent analysis. In summary, both performance measures and montage groups indicated that performance improved dramatically then saturated, suggesting that most subjects learned the Type-A solution effectively.

#### Two-digit addition arithmetic task

**Fig 5:**
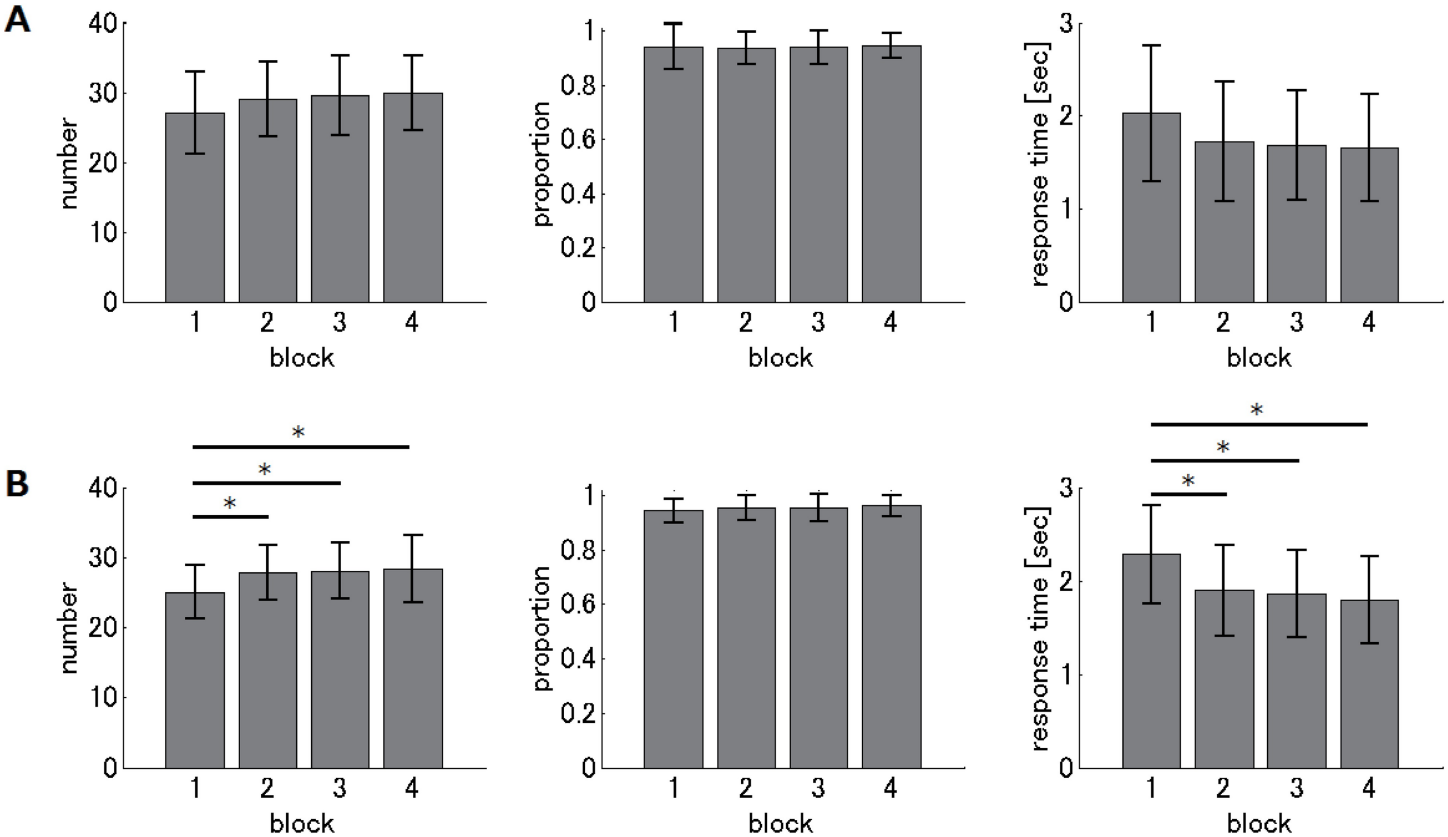
Results of the two-digit addition arithmetic task during the learning session. The left, middle, and right panels show results for the number of correct answers, proportion of correct answers and response time for correct answers, respectively. Each gray column represents the mean (across subjects) value for each block. Error bars represent standard deviations. Asterisks indicate significant (p < 0.05) differences between blocks. (A) The “bi-cephalic” group. (B) The “extra-cephalic” group.

Fig 5 shows changes in performance measures on the two-digit addition arithmetic task with the increased number of blocks in the learning sessions, for each montage group. For the “bi-cephalic” group (Fig 5A), one-way ANOVA revealed no significant effect of block on all performance measures (F_(3,124)_ = 1.62, p = 0.1871 for number of correct answers; F_(3,124)_ = 0.14, p = 0.9347 for proportion of correct answers; F_(3,124)_ = 2.3, p = 0.0807 for response time of correct answers), suggesting that performance saturated. For the “extra-cephalic” group (Fig 5B), one-way ANOVA revealed no significant effect of block on the proportion of correct answers (F_(3,119)_ = 0.72, p = 0.5406), and a significant effect of block on number (F_(3,119)_ = 4.36, p = 0.006) and response time (F_(3,119)_ = 6.34, p = 0.0005) of correct answers. The post-hoc Tukey’s HSD test revealed that both number and response time of correct answers in the first block were significantly different from those in other three blocks (p < 0.05) and that performance was not significantly different among the last three blocks (p > 0.05), suggesting that performance dramatically improved, then saturated. In summary, for all performance measures and montage groups, performance was not significantly different between the last two blocks, suggesting that most subjects became familiar with the two-digit addition arithmetic task.

### tDCS session

All subjects tolerated the stimulation, and none reported significant discomfort at the electrode sites or asked to stop the experiment because of side effects of the stimulation. The reported sensation of stimulation was stronger during active tDCS than sham sessions for 10 subjects (correct judgment), stronger during sham than active tDCS sessions for four subjects (incorrect judgment), and was no different between the two sessions for 10 subjects. A Fisher’s exact test yielded p > 0.12 for all tasks, suggesting that whether subjects correctly identified the difference between the tDCS sessions or not (correct judgment vs. others) did not alter the effects of tDCS (positive vs. negative).

#### Matchstick arithmetic task

Table 2 shows the percentage of subjects who identified the correct solution for each problem in the tDCS session described in the leftmost column. For the “bi-cephalic” group, the number of correct answers was greater in the active than sham tDCS sessions (positive tDCS effect) for eight subjects, whereas it was reduced in the active compared with sham tDCS sessions (negative tDCS effect) for four subjects. There was no difference between the two tDCS sessions (no tDCS effect) for 17 subjects. Paired samples t-tests revealed that the number of correct answers did not significantly differ between active and sham tDCS sessions (p = 0.26; mean (± SD) numbers of correct answers were 0.72 ± 0.53 and 0.59 ± 0.50 for active and sham tDCS sessions, respectively). The bias-corrected effect size (Hedges’ g) with lower and upper CIs was 0.26 (−0.25, 0.78).

**Table 2:** Results of the matchstick arithmetic task.

Session	% of subjects solving the problem			
	(N_solve / N_all)			
	“bi-cephalic”		“extra-cephalic”	
	Type-B	Type-C	Type-B	Type-C
**active tDCS only**	6.90	20.7	3.57	21.4
	(2/29)	(6/29)	(1/28)	(6/28)
**sham tDCS only**	0.00	13.8	0.00	14.3
	(0/29)	(4/29)	(0/28)	(4/28)
**both active and sham tDCS**	0.00	44.8	0.00	28.6
	(0/29)	(13/29)	(0/28)	(8/28)
**neither active nor sham tDCS**	93.1	20.7	96.4	35.7
	(27/29)	(6/29)	(27/28)	(10/28)

Values denote the percentage of subjects who found the correct solution in the tDCS session(s) described in the left column. The numbers in parentheses are the number of subjects who solved the problem (N_solve) over the total number of subjects (N_all). For example, the top right cell indicates that 21.4% of subjects (six subjects) in the “extra-cephalic” group identified the correct answer to Type-C problems only in the active tDCS session.

For the “extra-cephalic” group, seven subjects showed a positive tDCS effect, while four showed negative effect; 17 subjects did not show any tDCS effect. Paired samples t-tests revealed that the number of correct answers did not significantly differ between active and sham tDCS sessions (p = 0.38; mean [± SD] numbers of correct answers were 0.54 ± 0.51 and 0.43 ± 0.50 for active and sham tDCS sessions, respectively). The corrected effect size with lower and upper CIs was 0.21 (−0.32, 0.73).

For cases in which problems were solved correctly in both the active and sham tDCS sessions, tDCS may facilitate the speed of finding a solution. Thus, we compared response times for Type-C problems between active and sham tDCS sessions, for only 13 / 8 subjects (bi-cephalic / extra-cephalic) who correctly solved the problem in both sessions. It should be noted that no subjects found the right answer to the Type-B problems in both sessions (see Table 2). Paired samples t-test revealed no significant difference between active and sham sessions for both “bi-cephalic” (p = 0.41; mean [± SD] response times were 65.2 ± 72.7 s and 91.4 ± 73.7 s for active and sham tDCS sessions, respectively) and “extra-cephalic” (p = 0.80; mean [± SD] reaction times were 78.0 ± 54.4 s and 82.7 ± 43.1 s for active and sham tDCS sessions, respectively) groups. The bias-corrected effect sizes with lower and upper CIs were −0.35 (−1.12, 0.43) and −0.09 (−1.07, 0.89) for the “bi-cephalic” and “extra-cephalic” groups, respectively.

#### RAT task

Table 3 shows the percentage of subjects whose performance met the requirement described in the leftmost column. We first describe the results in the “bi-cephalic” group. For the number of correct answers, paired samples t-test revealed no significant difference between active and sham tDCS sessions (p = 0.43; mean [± SD] numbers of correct answers were 7.34 ± 2.24 for active tDCS sessions and 7.75 ± 1.93 for sham tDCS sessions). The bias-corrected effect size with lower and upper CIs was −0.19 (−0.68, 0.30). For the proportion of correct answers, a paired samples t-test revealed no significant difference between active and sham tDCS sessions (p = 0.41; mean [± SD] proportions of correct answers were 0.59 ± 0.12 for active tDCS sessions and 0.61 ± 0.11 for sham sessions). The bias-corrected effect size with lower and upper CIs was −0.20 (−0.69, 0.29).

**Table 3:** Results of the RAT task.

Requirements	% of subjects meeting the requirement			
	(N_meet / N_all)			
	“bi-cephalic”		“extra-cephalic”	
	number	proportion	number	proportion
**active tDCS > sham tDCS**	43.8	43.8	32.3	41.9
	(14/32)	(14/32)	(10/31)	(13/31)
**active tDCS = sham tDCS**	12.5	6.25	25.8	12.9
	(4/32)	(2/32)	(8/31)	(4/31)
**active tDCS < sham tDCS**	43.8	50.0	41.9	45.2
	(14/32)	(16/32)	(13/31)	(14/31)

Each value indicates the percentage of subjects whose performance measure (the number or proportion of correct answers) met the requirement described in the leftmost column. The numbers in parentheses are the number of subjects who met the requirement (N_meet) over the total number of subjects (N_all). For example, the top right cell indicates that, for 41.9% of subjects (13 subjects) in the “extra-cephalic” group, the proportion of correct answers was greater in active tDCS sessions than in the sham tDCS sessions.

Next, we discuss the results for the “extra-cephalic” group. For the number of correct answers, a paired samples t-test revealed no significant difference between active and sham tDCS sessions (p = 0.92; mean [± SD] numbers of correct answers were 7.06 ± 2.24 for active tDCS sessions and 7.10 ± 2.51 for sham tDCS sessions). The bias-corrected effect size with lower and upper CIs was −0.01 (−0.51, 0.48). For the proportion of correct answers, a paired samples t-test revealed no significant difference between active and sham tDCS sessions (p = 0.78; mean [± SD], proportions of correct answers were 0.58 ± 0.12 for active tDCS sessions and 0.59 ± 0.15 for sham sessions). The bias-corrected effect size with lower and upper CIs was −0.04 (−0.54, 0.45).

#### Two-digit addition arithmetic task

Table 4 shows the percentage of subjects whose performance met the requirement described in the leftmost column. We first describe the results of the “bi-cephalic” group. For the number of correct answers, a paired samples t-test revealed no significant difference between active and sham tDCS sessions (p = 0.51; mean [± SD] numbers of correct answers were 30.3 ± 5.75 for active tDCS sessions and 30.0 ± 4.81 sham tDCS sessions). The bias-corrected effect size with lower and upper CIs was 0.05 (-0.44, 0.54). For the proportion of correct answers, paired samples t-tests revealed no significant difference between active and sham tDCS sessions (p = 0.49; mean [± SD] numbers of correct answers were 0.97 ± 0.04 for active tDCS sessions and 0.97 ± 0.04 for sham tDCS sessions). The bias-corrected effect size with lower and upper CIs was 0.12 (−0.37, 0.61).

**Table 4:** Results of the two-digit addition arithmetic task.

Requirements	% of subjects meeting the requirement			
	(N_meet / N_all)			
	“bi-cephalic”		“extra-cephalic”	
	number	proportion	number	proportion
**active tDCS > sham tDCS**	34.4	37.5	29.0	32.3
	(11/32)	(12/32)	(9/31)	(10/31)
**active tDCS = sham tDCS**	18.8	31.3	19.4	16.1
	(6/32)	(10/32)	(6/31)	(5/31)
**active tDCS < sham tDCS**	46.9	31.3	51.6	51.6
	(15/32)	(10/32)	(16/31)	(16/31)

Each value indicates the percentage of subjects whose performance measure (the number or proportion of correct answers) met the requirement described in the leftmost column. The numbers in parentheses are the number of subjects who met the requirement (N_meet) over the total number of subjects (N_all). For example, the top right cell indicates that 32.3% of subjects (10 subjects) in the “extra-cephalic” group solved more problems in the active tDCS session than in the sham tDCS session.

Next we describe the results for the “extra-cephalic” group. For the number of correct answers, paired samples t-tests revealed no significant difference between active and sham tDCS sessions (p = 0.23; mean [± SD] numbers of correct answers were 29.0 ± 4.26 for active tDCS sessions and 29.6 ± 4.17 for sham tDCS sessions); the bias-corrected effect size with lower and upper CIs was −0.14 (−0.63, 0.36). For the proportion of correct answers, a paired samples t-test revealed no significant difference between active and sham tDCS sessions (p = 0.46; mean [± SD] numbers of correct answers were 0.96 ± 0.04 for active tDCS sessions and 0.97 ± 0.03 for sham tDCS sessions). The bias-corrected effect size with lower and upper CIs was −0.18 (−0.68, 0.32).

**Fig 6:**
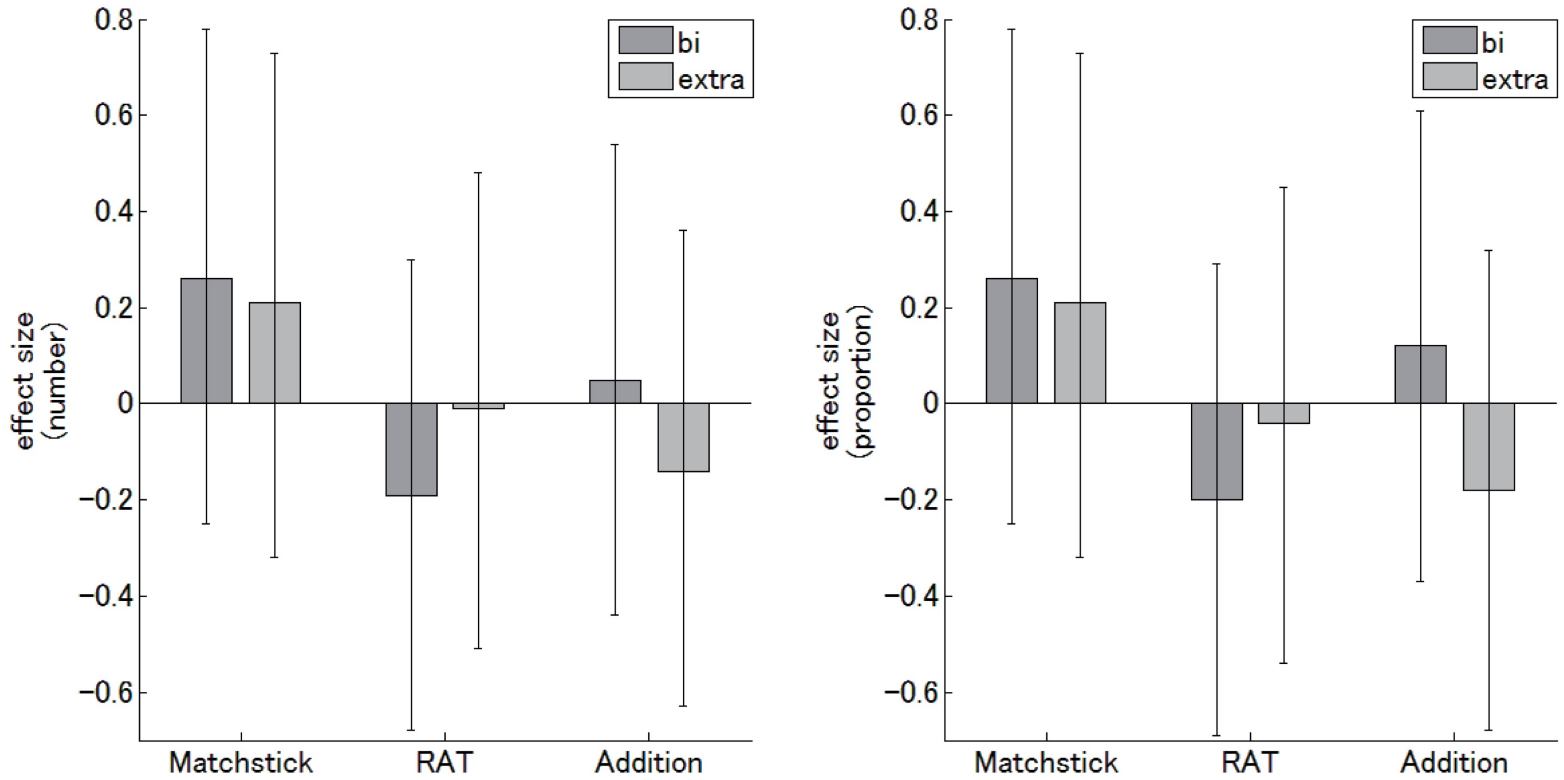
Results of all tasks during the test phase of the tDCS sessions. The left and right panels show the number and proportion of correct answers, respectively. The dark and light gray columns represent the effect sizes for the “bi-cephalic” and “extra-cephalic” montages, respectively. The error bars represent 95% confidence intervals.

Fig 6 summarizes effect sizes for all tasks. It can be seen that the effect size for the number of correct answers and the proportion of correct answers showed similar trends.

## Discussion

In the present study, we examined (1) whether anodal tDCS to the right ATL enhances verbal as well as non-verbal insights, (2) whether anodal tDCS to the right ATL facilitates non-verbal insight within as well as between subjects, and (3) specific effect of anodal tDCS to the right ATL on insight performance (and, as a result, the effects of cathodal tDCS to the left ATL on insight performance).

In the case of non-verbal insight, paired samples t-tests did not reveal any significant effects of tDCS, regardless of the electrode montage type. However, it is difficult to draw meaningful conclusions from the current data because there were only two problems, particularly considering that only three subjects across the two groups were able to solve one of the matchstick arithmetic tasks (Type-B problem). Regarding verbal insight, regardless of the electrode montage type, effect sizes were negative and failed to reach statistical significance in paired samples t-tests. These findings failed to reinforce previous neuroimaging studies showing the correlation between right ATL activity and verbal insight [7, 8, 9]. Regarding our first research question, this result indicates that, at least for verbal insight, anodal tDCS to the right ATL did not significantly enhance performance. Regarding the second question, it is difficult to draw conclusions because of a lack of statistical power.

For an “extra-cephalic” montage stimulating only the right ATL, the effect size was 0.21 for non-verbal insight, and approximately 0 for verbal insight. Regarding the third question, the results demonstrated that the specific effect of anodal tDCS of the right ATL on insight performance did not reach statistical significance in paired samples t-tests. In addition, there was little effect of cathodal tDCS to the left ATL on verbal insight performance, with no significant effects of tDCS observed for the “bi-cephalic” montage stimulating both ATLs and correspondingly small effect sizes.

### Discrepancy with previous findings

There are several possible reasons for the discrepancy between the current results and the findings of previous studies. As discussed above, the current study differed from previous experiments in terms of experimental design (i.e., within-subjects design), electrode montage type (i.e., “extra-cephalic” montage) and category of insight (i.e., verbal insight). The current results did not support our predictions, particularly regarding the experimental design. Because within-subjects designs typically have greater statistical power than between-subjects designs, we expected to find stronger effect sizes than previous studies. Below we discuss a range of possible factors that may have influenced this discrepancy.

First, differences in the details of the task may have affected the results. Regarding the matchstick arithmetic task, Chi and Snyder [10] used Roman numerals, whereas the current study used Arabic numerals. A difference in numerals may have caused differences in the number of possible changes from a one-digit number to another one-digit number (by moving only one stick). This may cause a difference in task difficulty; the greater the number of possible changes, the more difficult the task will become. In addition, Arabic numerals are common whereas Roman numerals are rare in Japan (as in many other countries). The difference in familiarity with the numerals used may have caused a difference in task difficulty. Considering that the effect of tDCS depends on task difficulty [32], the difference in numerals used may have impacted the effect of tDCS. Furthermore, the current study implemented a 4-minute time limit, compared with the 6-minute time limit used by Chi and Snyder [10]. The shorter time limit may have been insufficient to reveal the possible modulatory effects of tDCS, considering that only three participants across the whole group of participants were able to solve Type-B problems. Regarding the RAT task, previous neuroimaging studies reporting a correlation between right ATL activity and verbal insight performance [7, 8, 9] used a version of the RAT task (the CRA) consisting of English characters, whereas the current study used the Japanese version of the RAT task [23] consisting of kanji characters. Importantly, English characters are phonograms, whereas kanji or Chinese characters are not only phonograms but also ideograms. In addition to the language difference, our task also included distracters, unlike previous studies. In the standard RAT task used in previous studies [7, 8, 9], subjects’ semantic networks in the temporal lobes were freely activated, because the task did not include distracters. The RAT task with distracters, however, may interfere with the free activation of semantic processing. Thus, differences in the task details may have led to differences in task difficulty and neural processing involved in problem solving, which may have affected the effect of tDCS on task performance.

Second, in the condition combining verbal insight and the “bi-cephalic” montage, the cathode may have decreased activity in language-processing areas, resulting in a failure to enhance verbal insight performance. In the present study, the position of the stimulation electrode was determined based on the international 10–20 system. Thus, the stimulated regions may not have always been restricted to the target area, but could have also included adjacent areas due to inter-individual differences in cranial and brain anatomy [33, 34]. Moreover, the electrode surface area is relatively large (35 cm^2^ in the present study) and electrical currents exhibit diffusion through the scalp and cranium, further suggesting that tDCS might have stimulated additional adjacent cortical areas [15, 35, 36] even though the center of the stimulation electrode was placed directly above the intended area. Considering that language-processing areas are located in the LH temporal area, cathodal tDCS in the present study might have deactivated language-processing areas. This possibility may be supported by the finding that the effect size in the “bi-cephalic” montage condition was more negative than that in the “extra-cephalic” montage. Thus, cathodal tDCS may have impaired language processing, hindering the enhancement of verbal insight performance. Thus far, we have discussed our experimental results based on the assumption that cathodal tDCS has inhibitory effects. However, it is important to note that although cathodal tDCS has been shown to be inhibitory in most motor studies, this is not the case for cognitive studies, in which the question of whether cathodal tDCS has only inhibitory effects remains contentious [37]. Thus, it is difficult to draw firm conclusions about the effects of cathodal tDCS on insight from the current results.

### Future perspectives on tDCS for investigating insight

Although our results suggest that the right ATL is not a central locus of all types of insight, this does not imply that there is no such area. Four previous studies [12, 13, 14, 15] have suggested at least two candidates.

The first is the right TPJ. Goel and colleagues [15] found that anodal tDCS to the right TPJ together with cathodal tDCS to the left middle temporal gyrus (MTG) improved performance on a verbal insight task (riddles). This finding raises the question of whether anodal tDCS to the right TPJ can also improve performance on non-verbal (e.g., the matchstick task) insight problems and verbal (e.g., the RAT task) insight problems. The second candidate is the left DLPFC. Anodal tDCS to the left DLPFC has been associated with improvements in performance on another verbal insight task (the CRA test) [12, 13, 14]. According to Metuki and colleagues [13], insight problem solving consists of two processes: solution generation and recognition. The authors suggest that tDCS to the left DLPFC enhances recognition rather than generation. This proposal raises the question of whether the left DLPFC is also associated with solution recognition in solving non-verbal insight problems (e.g., the matchstick arithmetic task). Thus, it may be useful for future studies to test the effects of applying anodal tDCS to these two candidate areas.

## Conclusion

In the present study, we conducted tDCS experiments with an anode placed over the right ATL and a cathode over the left ATL (the bi-cephalic electrode montage), or with an anode over the right ATL and a cathode over the left cheek (the extra-cephalic reference montage), using a within-subjects design. During stimulation, subjects were required to perform verbal insight, non-verbal insight and non-insight (i.e., control) tasks.

We observed that tDCS did not significantly affect performance on either verbal or non-verbal insight problems, or non-insight problems, although the finding for non-verbal insight was inconclusive because of a lack of statistical power. This result was found for both “bi-cephalic” and “extra-cephalic” montages. These results failed to reinforce previous tDCS findings demonstrating a causal relationship between the right ATL and non-verbal insight using a between-subjects design [10, 11], or previous neuroimaging findings suggesting a correlational relationship between the right ATL and verbal insight [7, 8, 9].

We propose that anodal tDCS of right ATL did not affect verbal insight in the current study. However, it should be noted that the RAT may not be a comprehensive indicator of verbal insight, limiting the conclusions that can be drawn from the results. Instead, some researchers consider RAT task performance to be an indicator of complex verbal associative thought [12]. As described in the Materials and Method section, we used a modified version of the RAT task with distracters, to increase the likelihood that the problem is solved with insight. However, we did not directly examine whether the problem was solved with insight. Future studies will be required to overcome this limitation.

## Supporting information

S1 File Supplementary analyses and results. (DOCX)
