# Supplementary material for: Anodal transcranial direct current stimulation of the right anterior temporal lobe did not significantly affect verbal insight
Source: PLoS One. 2017 Sep 13;12(9):e0184749. doi: 10.1371/journal.pone.0184749 (PMC5597234; doi:10.1371/journal.pone.0184749)
Supplement: S1 File — (DOCX) [file pone.0184749.s001.docx]

**Supplementary Analyses and Results**

1. **Simulation with COMETS**

As described in the main text, we compared simulated current distributions between “bi-cephalic” and “extra-cephalic” montages. In all simulations, the anode and cathode electrode pads were 5 × 7 cm in size, and the injection currents of each pad were 1 mA and −1 mA, respectively. The electrode positions are shown in Figure 1B. We used the human head model embedded in the COMETS toolbox, which was extracted from the standard Montreal Neurological Institute (MNI) brain atlas [1].

As seen in S1 Fig, the cortical current distributions of both montages showed clear differences, particularly around the left temporal area. For the left hemisphere, tDCS with the “bi-cephalic” montage activated widespread temporal areas including the ATL and language-processing areas, whereas tDCS with the “extra-cephalic” montage generated little activity. For the right hemisphere, tDCS with both montages activated widespread temporal areas, including ATL.


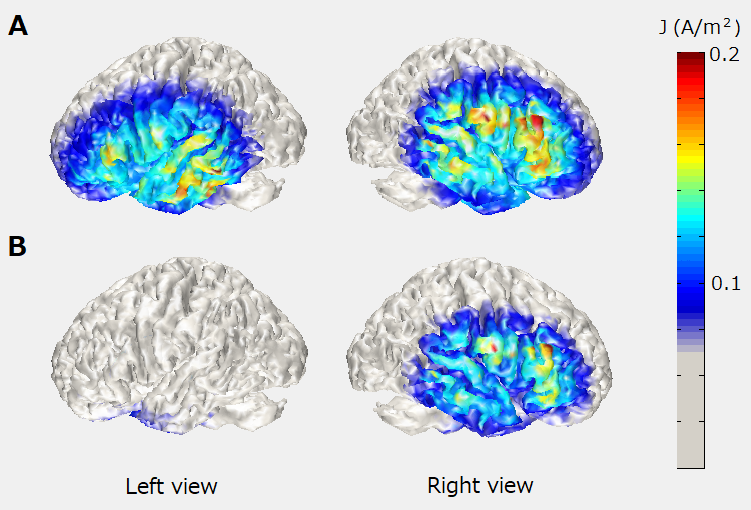


**S1 Fig.** **Simulated current distribution for “bi-cephalic” electrode montage (A) and “extra-cephalic” reference montage (B)**.

1. **Additional analyses of the effect of tDCS on insight**
   1. **Effect sizes for data combined between the two montages**

We calculated effect sizes for data combined between the two montage groups. In this analysis, we first tested whether data from the two montage groups could be combined or not, using mixed-design ANOVA with tDCS session (active vs. sham) as a within-subjects factor and electrode montage (bi-cephalic vs. extra-cephalic) as a between-subjects factor. After confirming that main effect of montage was not significant, we combined data between the two montage types and calculated effect sizes.

S1 Table summarizes the results of the mixed-design ANOVA using the MATLAB function mixed_between_within_anova.m in the MATLAB Central File Exchange by Matthew Johnson. Significant main effects of tDCS session and montage were not found. Moreover, the interaction between tDCS session and montage was not significant. These results support the validity of combining data between the two montage types.

**S1 Table. Results of mixed-design ANOVA.**

| **Task** | **Measure** | **Main effect**  **(session)** | **Main effect**  **(montage)** | **Interaction**  **(session×montage)** |
| --- | --- | --- | --- | --- |
| **Matchstick** | Number /  Proportion | F(1,224) = 2.13,  p = 0.15 | F(1,224) = 2.67,  p = 0.11 | F(1,224) = 0.03,  p = 0.86 |
| **RAT** | Number | F(1,248) = 0.52,  p = 0.48 | F(1,248) = 0.97,  p = 0.33 | F(1,248) = 0.38,  p = 0.54 |
| **RAT** | Proportion | F(1,248) = 0.68,  p = 0.41 | F(1,248) = 0.38,  p = 0.54 | F(1,248) = 0.24,  p = 0.63 |
| **Addition** | Number | F(1,248) = 0.23 ,  p = 0.64 | F(1,248) = 0.45,  p = 0.50 | F(1,248) = 1.87,  p = 0.18 |
| **Addition** | Proportion | F(1,248) = 0.02,  p = 0.89 | F(1,248) = 0.01,  p = 0.95 | F(1,248) = 1.03,  p = 0.31 |

Matchstick = matchstick arithmetic task; RAT = remote associates task; Addition = two-digit addition arithmetic task

S2 Fig shows the effect sizes with combined data for each task and performance measure. These results indicate that anodal tDCS to the right ATL had weak positive and negative effects on non-verbal and verbal insight problem solving, respectively, but the effects were not significant (a paired samples t-test: p = 0.1459 for both the number and proportion of correct answers in the matchstick arithmetic task; p = 0.4662 and 0.4064 for the number and proportion of correct answers in the RAT task, respectively; p = 0.6543 and 0.9045 for the number and proportion of correct answers in the two-digit addition arithmetic task, respectively). tDCS had a negligibly weak effect on mathematical non-insight problem solving.


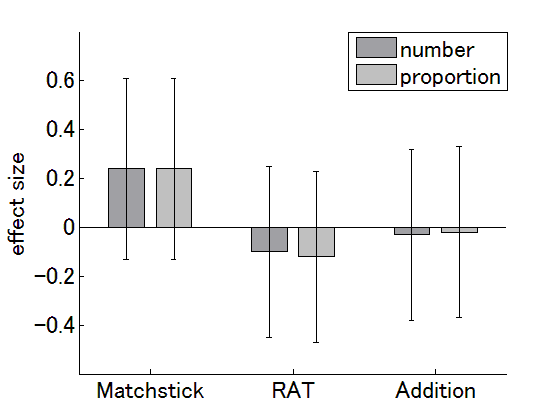


**S2 Fig. Effect sizes of data combined between the two montages.** The dark and light gray columns represent effect sizes for the number and proportion of correct answers, respectively. The error bars represent CIs. Note that, for the matchstick arithmetic task only, the number of correct answers was positively correlated with the proportion of correct answers, indicating that the effect sizes were the same between the two measures.

- 1. **Effect of tDCS on insight solution generation**

According to Metuki et al. [2], insight problem solving consists of solution generation and recognition processes. The right temporal lobe is thought to be involved in solution generation, whereas the left DLPFC is engaged in solution recognition [2]. If this is the case, anodal tDCS to the right ATL would be expected to activate the right temporal lobe and therefore facilitate solution generation. Thus, we speculated that improved solution generation would be reflected by an increased number of both correct and incorrect answers. To examine this possibility, we investigated whether tDCS affected the number of answers generated.

S3 Fig displays bar graphs of effect sizes with CIs for each case. In most cases, effect sizes were negligibly small. The effect size was moderate only in the matchstick task with the “bi-cephalic” montage. However, the difference between active and sham tDCS sessions was not significant (t-test, p = 0.07). These results suggest that both temporal lobes may not be involved in solution generation, inconsistent with the hypothesis proposed by Metuki et al. [2]. However, as described in the main text, the inhibitory effects of cathodal tDCS are still contentious. Therefore, further investigation in future studies is necessary to clarify this issue.


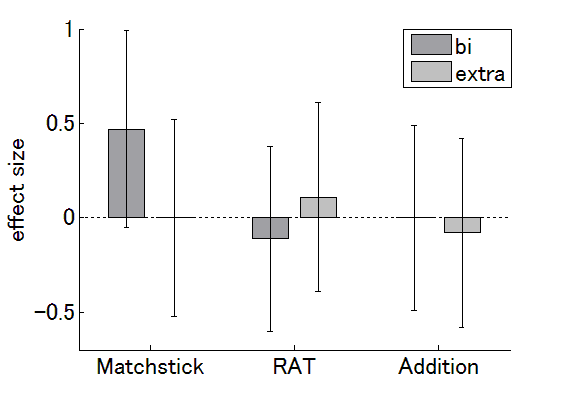
 **S3 Fig. Effect sizes for the numbers of answers.** The dark and light gray columns represent effect sizes for the number of answers with “bi-cephalic” and “extra-cephalic” montages, respectively. The error bars represent CIs. Matchstick = matchstick arithmetic task; RAT = remote associates task; Addition = two-digit addition arithmetic task

**2.3. Effect of tDCS on response times**

We also investigated whether tDCS affected response times (RTs). For both the matchstick arithmetic and the RAT tasks, we compared RTs of correct answers between the active and sham tDCS sessions.

A two-sample t-test revealed that p = 0.1971 for the matchstick arithmetic and p = 0.7931 for the RAT task, suggesting that tDCS did not significantly affect RTs for either insight type.

**2.4. Survival analysis**

In a previous study of the matchstick arithmetic task [3], a survival (time to event) analysis was used to compare whether there were any differences in time to event curves between the active tDCS group and the sham tDCS group. Thus, we conducted a similar analysis in the following way. First, for each problem, time to event curves were plotted using the Kaplan-Meier method. Then, we conducted a comparison of the curves between the active and sham tDCS sessions using the logrank test [3].

The logrank test revealed that the condition of stimulation did not have a significant effect on the time to event curves for either Type-B problems (p = 0.407) or Type-C problems (p = 0.471) for the “bi-cephalic” group. Similar results were found for the “extra-cephalic” group (p = 0.939 for Type-B problems and p = 0.785 for Type-C problems).

**3. Additional analyses of the effect of other factors on insight**

There is a possibility that factors other than tDCS affected performance on insight problems. Thus, we conducted several additional analyses.

**3.1. Effect of subject’s ability on performance**

It is well known that the effects of tDCS depend on a subject’s ability. Previous studies of motor control [4] and visual cognition [5] reported that low performers benefitted from stimulation, whereas high performers did not, most likely due to a “ceiling effect”. In the current study, we investigated whether the effects of tDCS depended on pre-learning (i.e., natural) ability. Moreover, we investigated whether the effects of tDCS depended on (1) post-learning ability and (2) amount of learning. These analyses were conducted only for the matchstick arithmetic task, because the learning session did not include the RAT task.

In these analyses, we adopted the proportion of correct answers as a performance measure. Importantly, in the learning session, the number of correct answers was positively correlated with the proportion of correct answers. We used performance in the first and last blocks of the learning session as measures of pre- and post-learning ability. Moreover, we used the difference in performance between the first and last blocks of the learning session as a measure of the amount of learning. For each montage group, subjects were divided into three groups according to tDCS effects (negative, no, and positive effects; see the Results section). The pre-/post-learning ability levels and amount of learning were then compared across tDCS effect groups (S4 Fig).


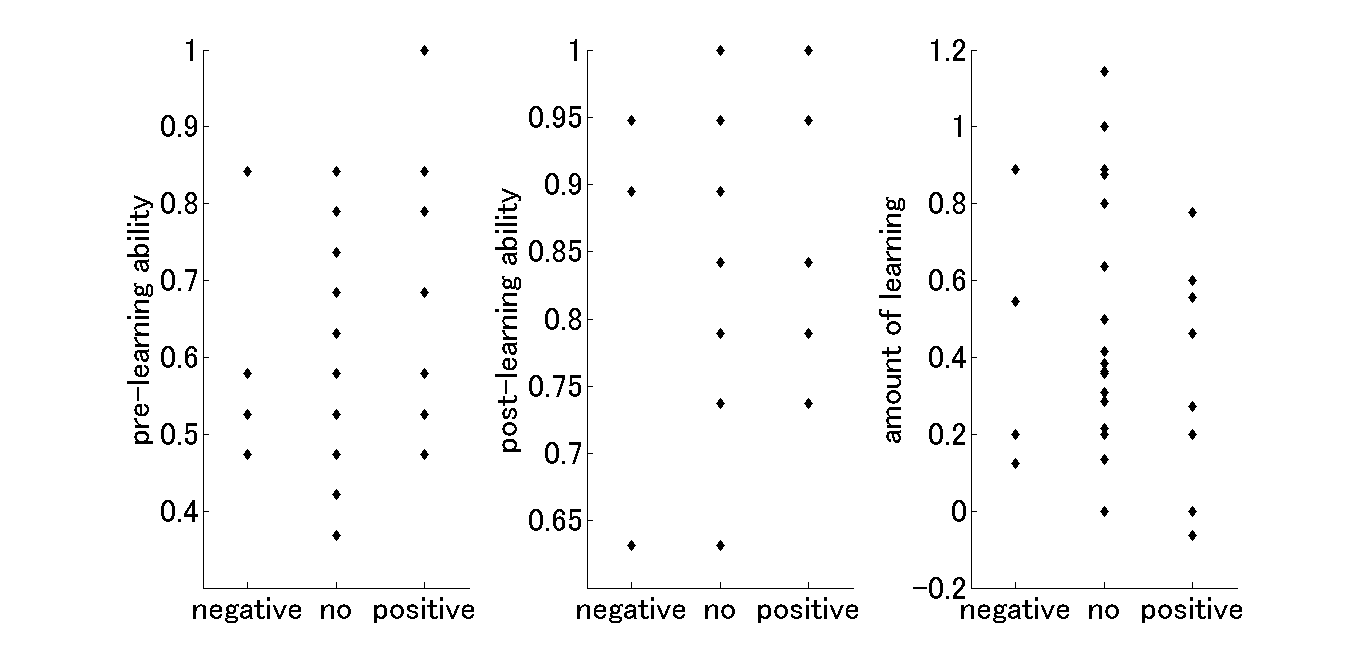


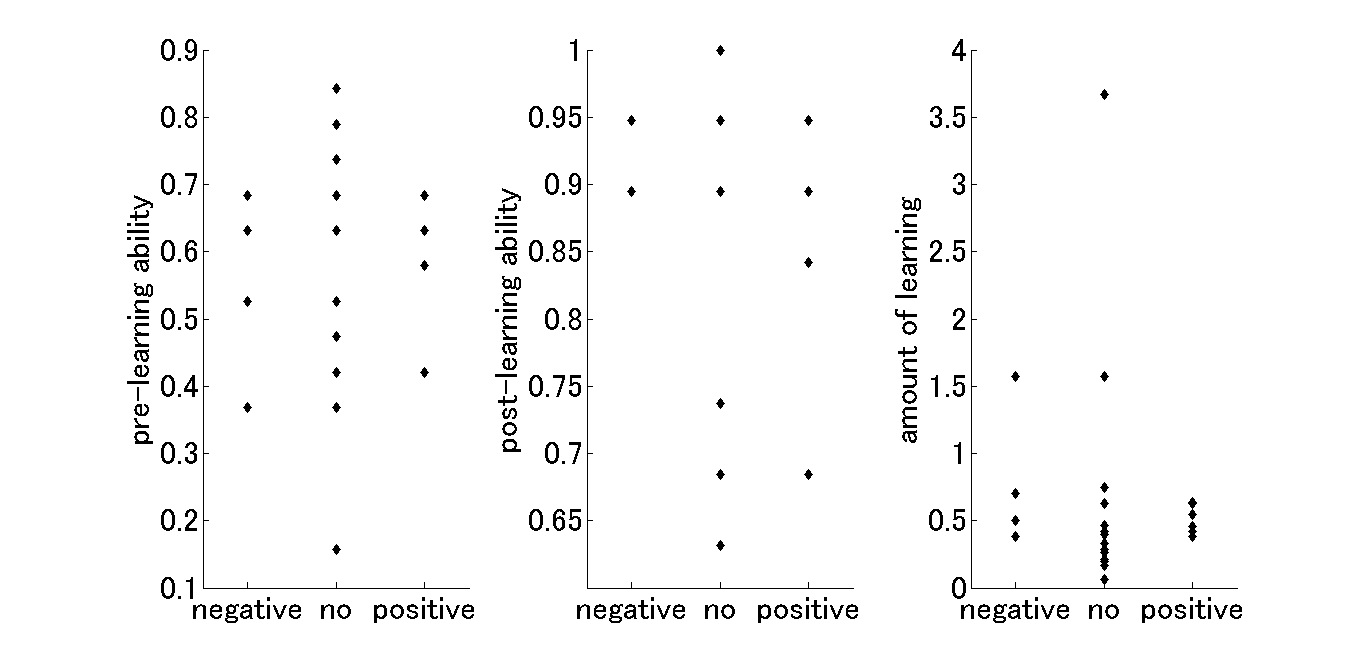


**S4 Fig. Matchstick arithmetic task performance during the learning session was compared between negative, no and positive tDCS effect groups.** The left, middle and right panels represent pre-learning ability (i.e., performance in the first block of the learning session), post-learning ability (i.e., performance in the last block) and amount of learning (i.e., difference in performance between the first and last blocks), respectively. Performance here refers to the proportion of correct answers. The upper and lower panels show the results for the “bi-cephalic” and “extra-cephalic” groups, respectively.

One-way ANOVA revealed that pre/post-learning ability and amount of learning did not differ across tDCS effect groups (for the “bi-cephalic” group, F[2, 26] = 0.54 and p = 0.5897 for pre-learning ability, F[2, 26] = 0.06 and p = 0.9453 for post-learning ability, and F[2, 26] = 0.58 and p = 0.5678 for the amount of learning; for the “extra-cephalic” group, F[2, 25] = 0.12 and p = 0.8855 for pre-learning ability, F(2, 25) = 0.86 and p = 0.4367 for post-learning ability, and F[2, 25] = 0.23 and p = 0.7994 for the amount of learning).

Thus, our data suggests that pre-learning (i.e., natural) ability did not alter the effects of tDCS, inconsistent with previous findings using other tasks [4, 5]. One possible reason for this discrepancy is related to the unique characteristics of the matchstick arithmetic task, because learning one solution strategy can impede solving a new problem that requires other solution strategies.

**3.2. Effect of mood on performance**

Subramaniam et al. [6] found that subjects who scored higher in positive mood solved more problems, and specifically solved more problems with insight, compared with subjects scoring lower in positive mood. We investigated whether performance on insight problems depended on positive mood. As described in the Materials and Methods section, we measured mood using the Japanese version of the PANAS at the beginning of each tDCS session. The PANAS consists of 10 positive and 10 negative items. For each item, subjects were instructed to give ratings on a 6-point scale. As a positive mood measure, Subramaniam and colleagues used the difference P – N, where P and N are the sums of the 10 positive and negative scores, respectively.

We adopted the number of correct answers as a measure of performance on the insight problems. In accord with a previous study by Subramaniam et al. [6], we conducted two analyses on these data. First, for both the matchstick arithmetic and RAT tasks, we drew scatter plots indicating the relation between performance (i.e., the number of correct answers) and positive mood (i.e., P – N value) and calculated the correlation coefficients. Second, we divided the data into three groups (high, middle, low) according to the P – N value, and compared performance (i.e., the number of correct answers) across the positive mood groups with one-way ANOVA. In both analyses, data were combined between tDCS sessions and between montage types.

S5 Fig shows the results of the first analysis. For both the matchstick and RAT tasks, the number of correct answers was not correlated with positive mood (r = −0.03, p = 0.74 for the matchstick arithmetic task; r = 0.02, p = 0.82 for the RAT task).


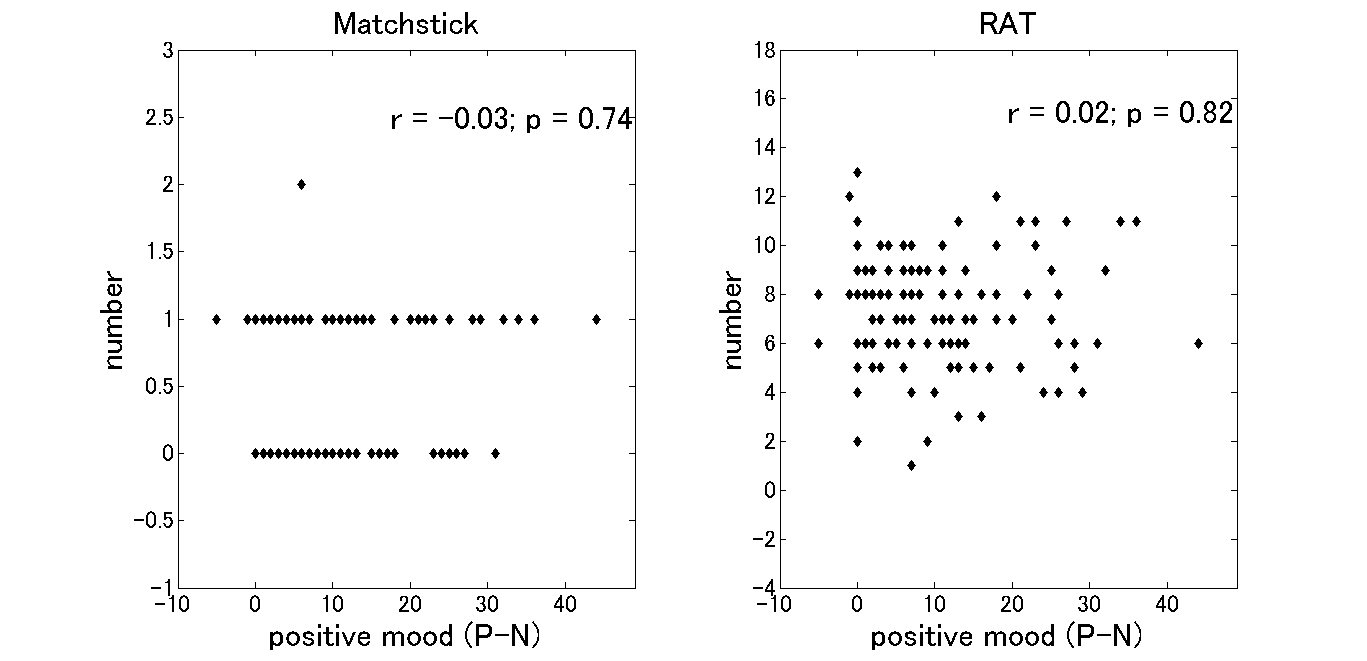


**S5 Fig. Scatterplots showing the relationship between the number of correct answers and positive mood (P – N).** Data were combined between the two montage types and between tDCS sessions. Results for the matchstick arithmetic and RAT tasks are shown in the left and right panels, respectively.

S6 Fig shows the results of the second analysis. For both the matchstick arithmetic and RAT tasks, one-way ANOVA revealed that the number of correct answers did not differ across positive mood groups (F[2, 111] = 1.33, p = 0.2688 for the matchstick arithmetic task; F[2, 123] = 0.68, p = 0.5064 for the RAT task).


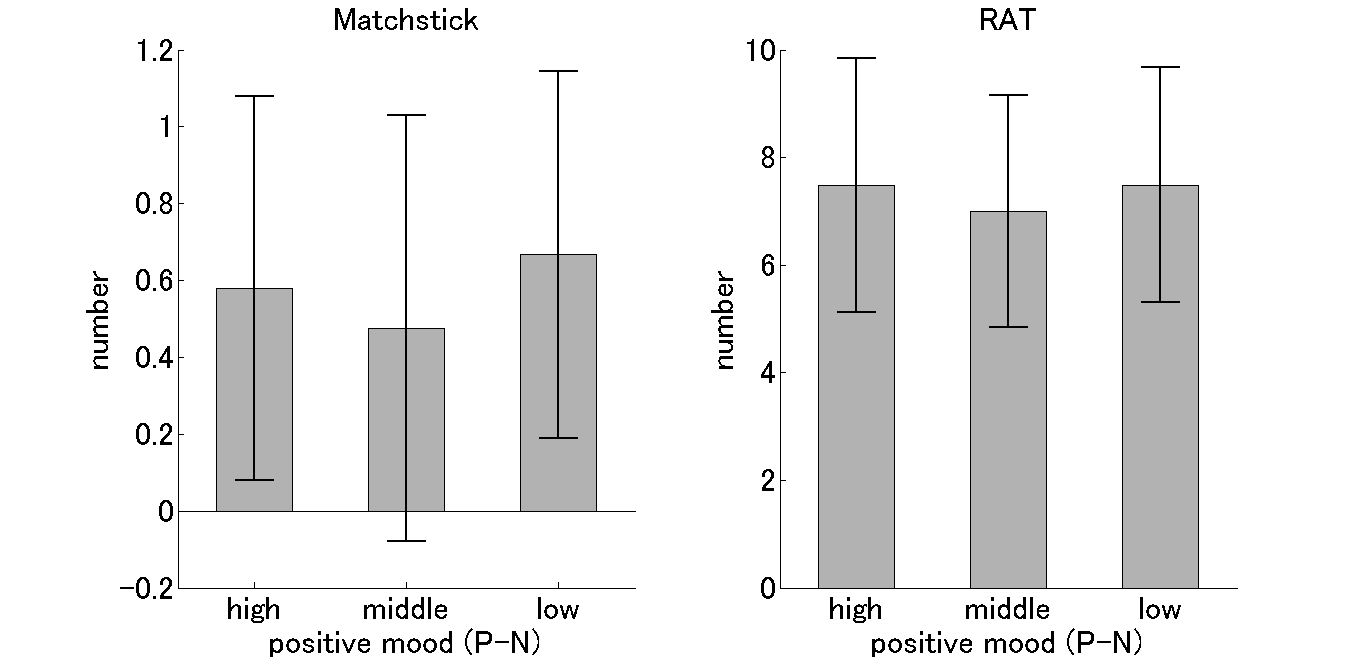


**S6 Fig. Numbers of correct answers for high, middle and low positive mood groups.** Each gray column represents the number of correct answers averaged within each mood group. The error bar~~s~~ represent standard deviations. Results for the matchstick arithmetic and RAT tasks are shown in the left and right panels, respectively.

Thus, the current results indicate that verbal as well as non-verbal insight were unaffected by positive mood. The former result is inconsistent with a previous finding reported by Subramaniam et al. [6]. This discrepancy in mood effects may be related to differences in task between the two studies. First, for both the RAT task and PANAS questionnaire, the previous study used the original (i.e., English) versions, whereas the current study used Japanese versions. In addition, our data may have included non-insight solutions as well as insight solutions, because we did not ask subjects to report directly which strategy (i.e., insight vs. non-insight) they used to achieve solutions. In contrast, the previous study included only insight solutions. Thus, further studies are required to determine whether positive mood affects insight.

**References**

1. Jung Y, Kim J, Im C. A MATLAB toolbox for simulating local electric fields generated by transcranial direct current stimulation (tDCS). Biomed Eng Lett. 2013; 3: 39-46.

2. Metuki N, Sela T, Lavidor M. Enhancing cognitive control components of insight problems solving by anodal tDCS of the left dorsolateral prefrontal cortex. Brain Stimul. 2012; 5: 110-115.

3. Chi RP, Snyder AW. Facilitate insight by non-invasive brain stimulation. PLoS ONE. 2011; 6: e16655.

4. Furuya S, Klaus M, Nitsche MA, Paulus W, Altenmuller E. Ceiling effects prevent further improvement of transcranial stimulation in skilled musicians. J Neurosci. 2014; 34: 13834-13839.

5. Tseng P, Hsu TY, Chang CF, Tzeng OJL, Hung DL, Muggleton NG, et al. Unleashing potential: Transcranial direct current stimulation over the right posterior parietal cortex improves change detection in low-performing individuals. J Neurosci. 2012; 32: 10554-10561.

6. Subramaniam K, Kounios J, Parrish TB, Jung-Beeman M. A brain mechanism for facilitation of insight by positive affect. J Cogn Neurosci. 2009; 21: 415-432.
